# A Multimodal Dual-Stream Framework for Sheep Behavior Recognition Using Skeletal and Local Visual Fusion

**DOI:** 10.3390/ani16172759

**Published:** 2026-09-02

**Authors:** Chuanzhong Xuan, Junze Jia, Suhui Liu, Zhaohui Tang

**Affiliations:** 1 College of Mechanical and Electrical Engineering, Inner Mongolia Agricultural University, Hohhot 010018, China; xcz@imau.edu.cn (C.X.); jiajunze@emails.imau.edu.cn (J.J.); lsh@emails.imau.edu.cn (S.L.); 2 Inner Mongolia Autonomous Region Engineering Research Center for Intelligent Equipment in Forage and Feed Production, Inner Mongolia Agricultural University, Hohhot 010018, China

**Keywords:** behavior recognition, keypoint detection, dual-stream fusion, skeletal kinematics, YOLOv11m-Pose

## Abstract

Intelligent sheep behavior monitoring is vital for modern husbandry, but faces severe challenges in natural pastures due to high-density flock occlusion. Traditional 2D skeleton-based networks often suffer from depth ambiguity and feature collapse, misclassifying static tremors as dynamic displacement. To overcome this, we propose a robust multimodal dual-stream framework using skeletal and local visual fusion. The architecture features an upstream spatial perception stage utilizing YOLOv11m-Pose. To reduce annotation costs and improve robustness, we introduce an Active Hard-Example Mining mechanism, explicitly retaining difficult samples with severe overlapping or edge truncation. For downstream behavioral decisions, a multimodal dual-stream architecture processes the targets. The Spatio–Temporal Kinematic Stream employs a Kinematic Denoising Engine, incorporating a 1D Gaussian filter and displacement dead-zone gate to purify 2D coordinates before feeding them into a BiLSTM network. Concurrently, the Spatial Visual Stream uses a ResNet-50 backbone on cropped RGB patches to capture essential spatial context, addressing the limitations of pure coordinates. Finally, a weighted Softmax layer integrates both streams. Experiments on a complex real-world dataset validate this approach. A baseline kinematic-only model achieved just 69.05% overall accuracy and 68.18% walking precision. In contrast, our dual-stream fusion network achieved 93.26% overall accuracy, elevating walking precision to 97.14% and the eating F1-score to 94.29%. By effectively decoupling similar static and dynamic behaviors, this study demonstrates the indispensability of local visual features, establishing a high-precision baseline for smart livestock monitoring.

## Simple Summary

Monitoring the daily behaviors of sheep, such as standing, walking, and eating, is essential for managing their health and welfare in modern smart farms. However, automatically recognizing these behaviors in natural pastures is highly challenging because sheep often crowd together, blocking the camera’s view. Traditional artificial intelligence methods that only track the animals’ body joints often struggle in these situations, misinterpreting slight bodily twitches as actual walking due to a lack of environmental context. To solve this, we developed a novel artificial intelligence system that simultaneously analyzes both the movement of the sheep’s skeleton and the surrounding visual background. By combining these two streams of information, our system can accurately distinguish between similar behaviors even when the sheep are heavily obscured. Tested on real-world farm video data, our method achieved a high overall accuracy of over 93%, significantly reducing false alarms. This technology offers a reliable, non-contact solution for continuous sheep monitoring, supporting better animal welfare and more efficient farm management.

## 1. Introduction

**Figure 1:**
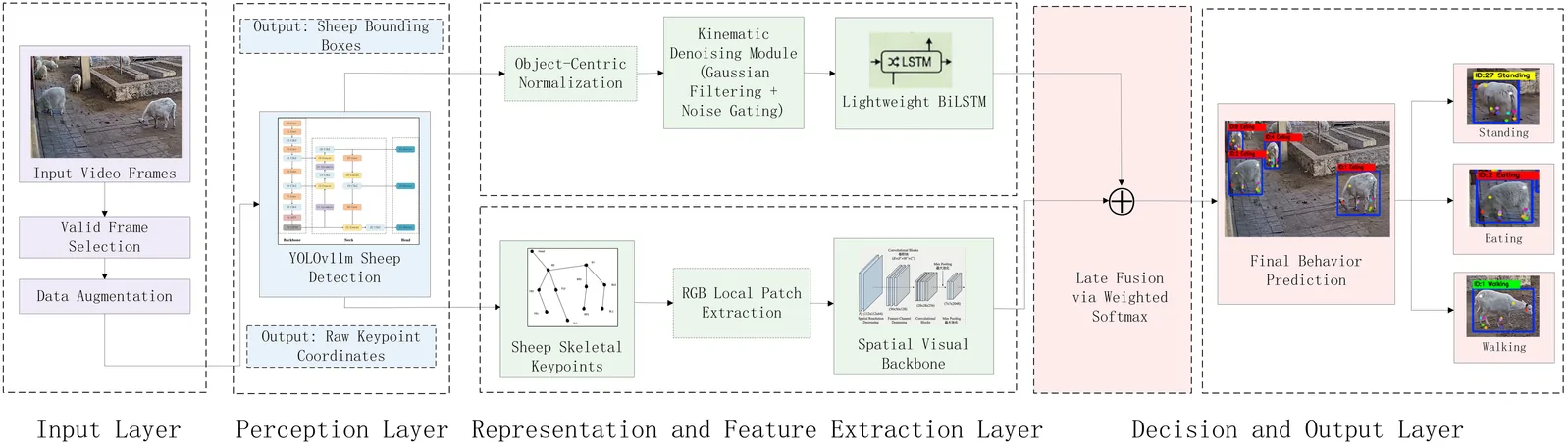
Overall method flow framework diagram.

With the rapid advancement of large-scale, intensive, and intelligent livestock farming, behavior recognition technologies underpinned by machine vision and deep learning have emerged as a critical technical foundation for smart farm development [1]. Among major livestock species, sheep are of particular importance, as their growth status, health condition, and daily behavioral patterns directly influence farming efficiency, disease prevention, and animal welfare [2]. Consequently, the deployment of non-contact, automated technologies for the continuous monitoring and precise behavioral analysis of sheep holds substantial practical significance and application value in precision livestock farming [3]. However, under natural grazing and housing conditions, sheep flocks typically exhibit dense spatial distributions, severe inter-individual occlusion, high phenotypic similarity, and complex behavioral postures, which pose formidable challenges to conventional detection and recognition approaches [4]. Achieving robust individual identification and accurate classification of key behaviors under such demanding real-world scenarios remains a pressing scientific challenge in animal husbandry engineering and agricultural informatization [5,6,7]. Traditional monitoring methods, including manual inspections, wearable sensors [8,9], and early visual algorithms relying on background modeling, are severely hindered by labor intensity, high deployment costs, animal stress, and a lack of robustness against multi-target occlusion and lighting variations [10]. While recent advancements have explored 3D reconstruction and multi-camera setups for detailed behavioral monitoring, these approaches present significant practical limitations. They typically require rigorous spatial calibration, incur high hardware and deployment costs, and are highly sensitive to the unstructured and dynamic lighting conditions inherent in natural pastures, making them difficult to scale in commercial farming. In recent years, deep learning has established a novel pathway for the non-contact behavioral analysis of individual sheep via object detection and pose estimation [11]. The primary challenge in this domain is ensuring accurate and robust keypoint detection under complex pastoral environments characterized by occlusion, illumination variations, and dense distributions [12,13]. To address this, recent studies have introduced Transformer-enhanced networks (e.g., VHR-BirdPose [14], DepthFormer [15]) and spatial-temporal modeling [16] to improve general keypoint detection. Furthermore, sheep-specific applications have explored customized frameworks, including YOLO-BCD for occluded scenarios [17], machine learning on keypoint-derived measurements [18], and 3D convolutions for fine-grained behaviors [19]. Although skeletal features exhibit superior robustness compared to bounding boxes [20,21], existing methods encounter three fundamental challenges when applied to continuous video streams in real-world farms: (1) detection failures and feature loss under severe inter-individual occlusion [22]; (2) temporal noise and spatial jitter inherited from upstream object detection [20,23]; (3) dimensional limitations of pure 2D coordinates, where the lack of depth and visual context conflates static jitter with true dynamic displacement, leading to false-positive errors [24,25]. To systematically address these limitations, this paper proposes a highly robust dual-stream fusion framework integrating visual context with skeletal dynamics. In the upstream stage, an active filtering mechanism based on hard-example priors [23] enables the YOLOv11m-Pose network to achieve occlusion-robust skeleton capture at a reduced annotation cost. In the downstream decision stage, a multimodal architecture is employed: a spatio–temporal dynamics stream (comprising a denoising engine and BiLSTM) effectively eliminates temporal jitter, while a spatial visual stream (utilizing a ResNet-50 backbone) compensates for the depth deficiencies of 2D coordinates [24,25]. Ultimately, a weighted Softmax decision layer fuses these modalities to facilitate highly robust behavior classification in complex scenes. The overall method workflow is illustrated in Figure 1.

As depicted in Figure 1, the proposed framework operates through a systematic pipeline comprising an upstream perception module and a downstream multimodal fusion module. Initially, continuous video sequences are fed into the YOLOv11m-Pose network, which is driven by an active filtering mechanism to robustly detect individual sheep and extract corresponding 2D skeletal keypoints alongside bounding boxes, effectively overcoming dense occlusions. Subsequently, the extracted data is routed into two parallel branches. In the spatio–temporal kinematic stream, the raw 2D coordinates are purified by a Kinematic Denoising Engine—incorporating a 1D Gaussian filter and a displacement dead-zone gate—to eliminate spatial jitter before temporal sequence modeling via a BiLSTM network. Concurrently, in the spatial visual stream, RGB patches cropped using the generated bounding boxes are processed by a ResNet-50 backbone to extract rich visual context and relative background displacements. Finally, the high-level semantic features from both modalities are concatenated and evaluated by a weighted Softmax classifier, which outputs the definitive behavioral decision. This end-to-end pipeline effectively bridges the gap between raw visual inputs and high-precision behavioral semantics, laying the foundation for the detailed methodologies discussed in the subsequent sections.

## 2. Materials and Methods

### 2.1. Data Acquisition and Dataset Construction

**Figure 2:**
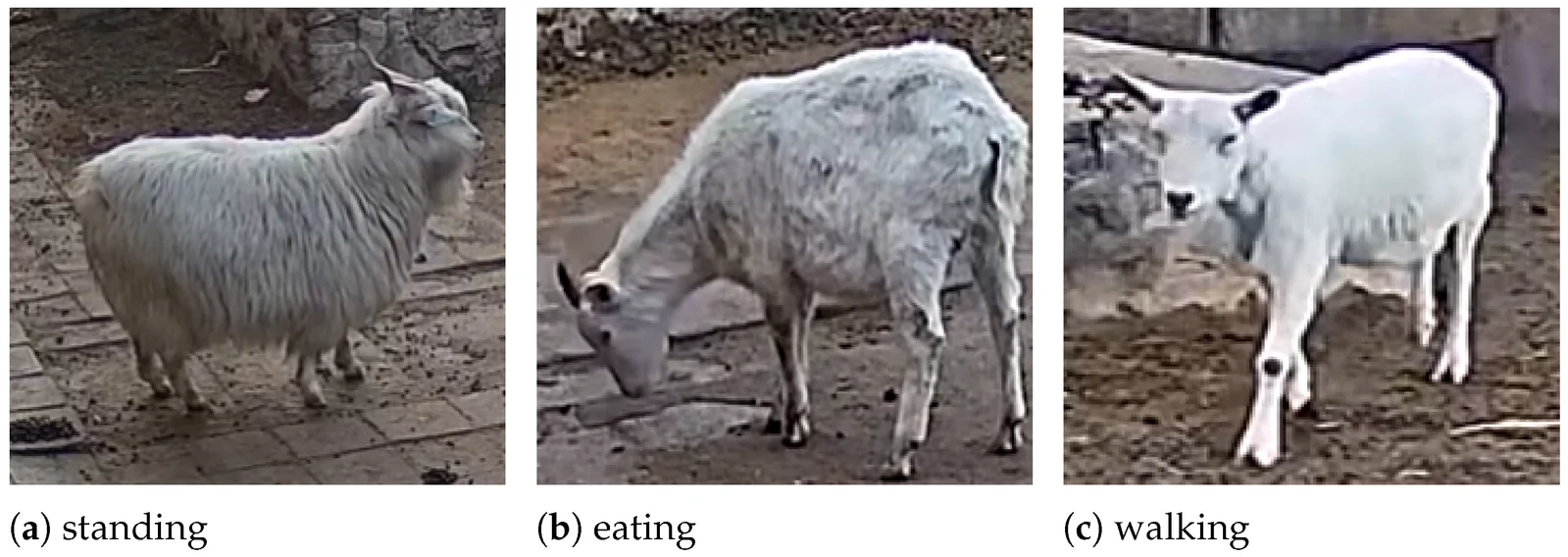
Diagram of different behaviors of sheep.

Data was collected between January and March 2025 at a commercial sheep farm in Hohhot, using a PoE camera (2560 × 1920, 30fps). The 40-h video corpus captured approximately 200 sheep across three primary behaviors: standing, eating, and walking (Figure 2). The dataset was collected from a flock of approximately 200 Small-tailed Han sheep, a common breed in northern China, consisting predominantly of adult sheep aged 1.5 to 3 years. All collected data was transmitted in real time to a central server via a PoE network interface for storage and backup. To ensure efficient data transmission and processing, video data is saved in high-definition MP4 format and is uniquely associated with a timestamp, facilitating subsequent data annotation and behavior recognition. The dataset was partitioned into training (80%) and testing (20%) sets. Crucially, a strict temporal isolation protocol was enforced between the two sets to prevent temporal autocorrelation leakage, thereby ensuring an unbiased evaluation of the model’s true generalization capacity. To prevent temporal information leakage and avoid overestimating generalization performance, the dataset split was performed strictly at the continuous video sequence level. Specifically, videos recorded during the first 25 days of the collection period were assigned exclusively to the training and validation sets, while continuous video segments from the final 5 days were reserved solely for the testing set. This guarantees that sequential frames from the same behavioral event do not overlap across subsets.

**Figure 3:**
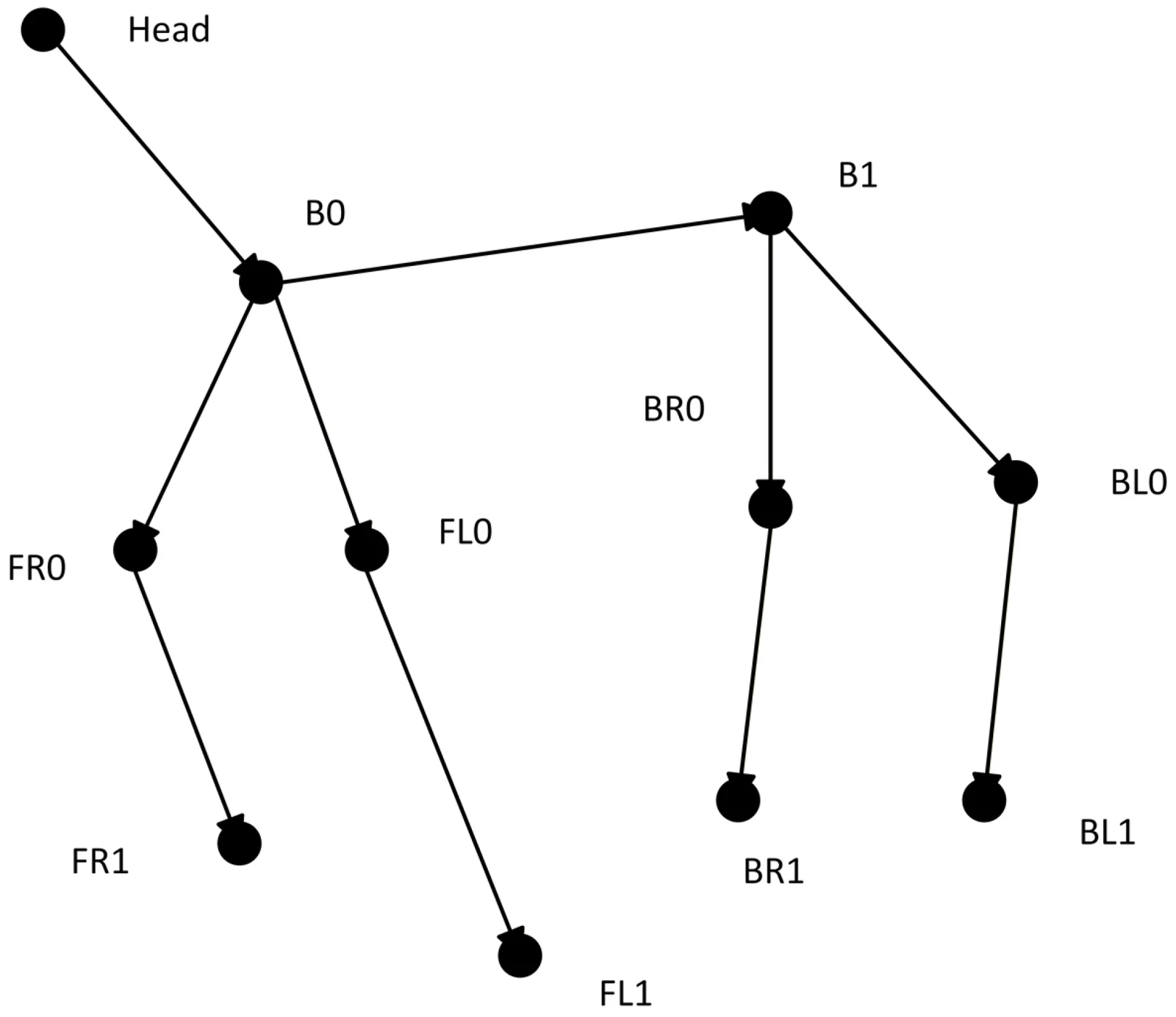
Schematic diagram of key skeletal points in sheep.

Before studying sheep pose extraction methods, it is crucial to establish the key skeletal points and their connection relationships to construct a complete skeletal model [26]. Through an in-depth analysis of sheep skeletal features and organ locations, 11 key points and 10 pairs of connection relationships were designed to comprehensively capture the sheep’s pose characteristics [27]. As illustrated in Figure 3, points 1–11 correspond to the head, neck, hindquarters, left forelimb joint, left forelimb hoof, right forelimb joint, right forelimb hoof, left hindlimb joint, left hindlimb hoof, right hindlimb joint, and right hindlimb hoof, respectively.

To meet the multi-level task requirements of upstream spatial perception and downstream behavior classification, this study strictly decoupled data annotation into distinct physical stages [28]. In natural grazing scenarios, uniform frame sampling yields redundant, low-complexity data, causing prohibitive annotation costs and network overfitting. To overcome this, we adopted an active screening mechanism guided by a Hard-Example Prior. Rather than blind sampling, a semi-automated, two-stage filtering procedure was applied to the raw video corpus to systematically discard isolated, unobstructed sheep and explicitly retain frames exhibiting complex physical challenges.

Initially, approximately 144,000 raw frames were extracted at 1 fps. An automated bounding-box overlap filter was then applied to discard scenes with isolated individuals, retaining only frames where the Intersection over Union between any two bounding boxes exceeded 0.3. This automated step reduced the dataset to approximately 18,500 candidate frames. Subsequently, a manual qualitative screening was conducted by trained annotators. The explicit objective criteria for retention included severe inter-individual limb occlusion, target truncation by the image boundary, and extreme posture variations. This rigorous manual refinement effectively enriched the dataset with complex scenarios, ultimately yielding 3358 high-value hard-example targets. Using Labelme software, these 3358 individual challenging sheep targets were extracted and finely annotated in strict accordance with the established topological order, covering various complex poses: standing (1072), eating (1106), and walking (1180). This perception dataset was designed to drive the YOLO-based pose-aware backbone, compelling it to infer missing limb relationships via global anatomical priors rather than relying solely on visible pixels.

Subsequently, leveraging the trained upstream model, we constructed a downstream behavioral representation dataset comprising 2089 valid action instances (697 standing, 700 walking, and 692 eating instances). This dataset automatically captured long-term spatio–temporal coordinates from continuous video streams while simultaneously extracting local color slices containing real-world backgrounds, providing a rigorous data loop for evaluating the dual-stream fusion architecture.

### 2.2. Proposed Multimodal Dual-Stream Framework

To achieve robust recognition of individual sheep postures in group-rearing environments, while accounting for practical farming scenarios, this paper selects standing, eating, and walking as the primary target categories. Drawing inspiration from recent advancements in spatio–temporal feature sharing for animal monitoring [29], this paper proposes a multimodal dual-stream recognition framework. Architecturally, the system is decoupled into three core modules: an upstream spatial perception network for target localization, a downstream spatio–temporal dynamic stream utilizing coordinate tracking and dynamic denoising, and a concurrent spatial visual stream based on image patches and scene context features. Ultimately, behavioral classification decisions are executed through a weighted late-fusion network.

Extracting a highly robust sequence of spatial keypoints is a prerequisite for constructing reliable skeletal dynamic features. To identify a perception network with optimal keypoint localization capabilities for heavily occluded pastoral environments, we directly evaluated four baseline models building upon recent successful deployments of YOLO-pose architectures [30]. The comparative results based on the aforementioned perception dataset are summarized in Table 1.

As quantitatively summarized in Table 1, YOLOv11m-Pose was selected as the upstream backbone network due to its optimal balance between computational efficiency and high-precision localization. Since dense visual computation entails significant computational overhead, this study retains a purely kinematic branch to serve as the baseline feature representation for temporal motion. For the set of 11 two-dimensional keypoint coordinates $P_{t}=\left\{p_{t,1},\ldots ,p_{t,11}\right\}$ of the target sheep in frame *t*, this paper constructs an independent kinematic reconstruction and denoising engine.

To eliminate the translation bias introduced by the target’s absolute position within the frame, we calculate the topological centroid $C_{t}$ for frame *t*, transform the coordinates into an ego-centric reference system, and perform extremum normalization:

(1) $$C_{t}=\frac{1}{11}\sum_{j=1}^{11}p_{t,j}$$

(2) $${\hat{p}}_{t,j}=\frac{p_{t,j}-C_{t}}{\max(|p_{t,j}-C_{t}|)+\epsilon }$$

where $p_{t,j}$ indicates the raw pixel coordinates of the *j*-th keypoint, and ϵ is a smoothing constant introduced to prevent division by zero (set to $1\times 10^{-5}$).

After the removal of RGB textures, minor high-frequency spatial jitters originating from the pose perception network are prone to triggering false-positive misclassifications. To sever this error propagation, we introduce a one-dimensional Gaussian low-pass filter (σ=2.0) along the temporal axis. Concurrently, the first-order raw kinematic derivative $V_{raw,t}$ is computed, and a dynamic displacement dead-zone (threshold τ=0.02) is designed. If the inter-frame relative displacement does not exceed 2% of the normalized scale, it is mathematically truncated:

(3) $$V_{t}=\left\{\begin{array}{cc} V_{raw,t}, & |V_{raw,t}|\geq \tau \\ 0, & |V_{raw,t}|<\tau \end{array}\right.$$

**Figure 4:**
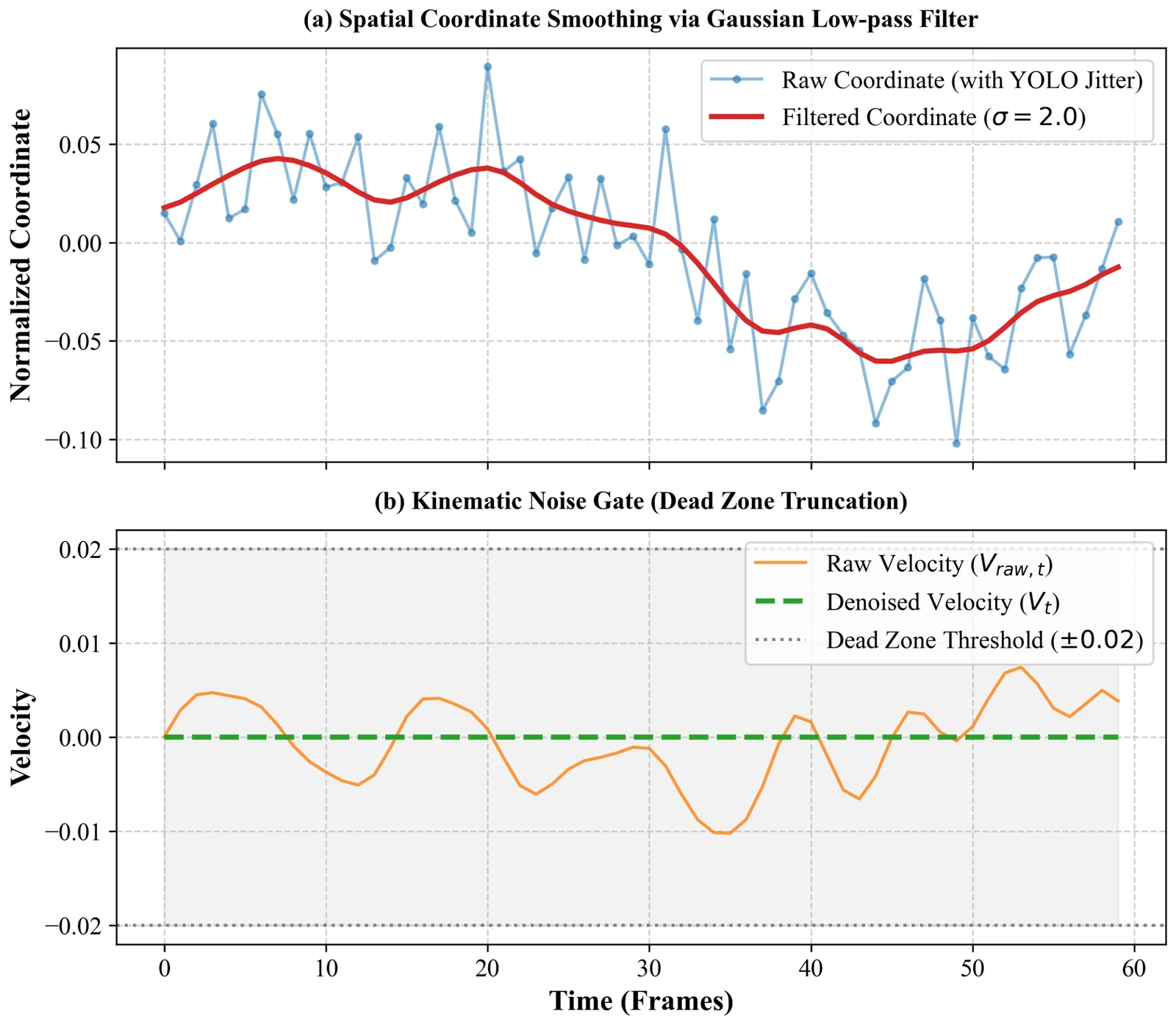
Mechanism of 1D signal smoothing and dead zone truncation in the kinematic denoising engine.

The displacement dead-zone threshold was determined empirically through a statistical analysis of the physiological micro-movements of the sheep. Observations indicated that involuntary movements, such as breathing tremors or slight head shifts during static standing, typically generated bounding box centroid displacements of less than 2% relative to the normalized scale. Consequently, setting the threshold to 0.02 effectively filters out these high-frequency physiological noises without discarding true dynamic walking displacements.To handle missing, unreliable, or anatomically abnormal joint coordinates caused by severe occlusion or physical anomalies, the Kinematic Denoising Engine incorporates a temporal imputation mechanism. When a keypoint’s detection confidence score falls below 0.2, the system applies temporal linear interpolation across adjacent frames to estimate the missing coordinate before executing the 1D Gaussian smoothing. This strategy prevents abrupt spatial jumps from being misclassified as dynamic motion, thereby enhancing the model’s robustness under extreme boundary conditions. The physical denoising mechanism is illustrated in Figure 4. The purified coordinates $P_{t}$ and velocity $V_{t}$ are concatenated into a 44-dimensional compact feature vector. Following temporal alignment, this vector is fed into a lightweight Bidirectional Long Short-Term Memory (BiLSTM) network, which outputs the classification log-probability $L_{kinematic}$ for the kinematic stream.

Scene context awareness relying exclusively on two-dimensional topological coordinates frequently suffers from depth loss and feature collapse, particularly when the target undergoes radial motion. To mitigate these physical limitations, this study introduces a spatial visual stream. Localized RGB patches that encompass both the target individual and the surrounding pastoral background are systematically resampled into standardized 224×224 pixel tensors. A ResNet-50 backbone, pre-trained on ImageNet, is employed as the primary visual feature extraction network. Through deep 2D convolutions, this stream effectively captures morphological variations in wool texture and relative background displacement. Ultimately, the network outputs the classification log-probability $L_{rgb}$. Utilizing weights pre-trained on ImageNet for the visual stream significantly impacts the model’s performance by providing highly robust generalized feature extractors for basic edges and textures. This initialization not only accelerates the convergence rate during training but also acts as a powerful regularizer, preventing the network from overfitting to the limited localized RGB patches of the pastoral background. The selection of ResNet-50 as the visual backbone was determined by a critical trade-off between empirical performance and inference latency for edge deployment. While deeper architectures such as ResNet-101 can capture more complex hierarchical representations, preliminary evaluations indicated that they introduced severe computational overhead without proportional gains for our localized 224×224 RGB patches. Specifically, deploying ResNet-101 marginally improved the overall F1-score by less than 0.5% but caused the inference speed to drop precipitously from 32.4 FPS to approximately 19.5 FPS. Given that real-time continuous monitoring in smart farming strictly requires maintaining inference speeds above 30 FPS, ResNet-50 represents the optimal Pareto frontier for this dual-stream architecture, balancing robust feature extraction with essential computational efficiency. To leverage the complementary advantages of multi-modal features, this study implements a late-fusion mechanism at the decision level. The system first utilizes a Softmax function to normalize the raw logits from both streams into valid probability distributions. Subsequently, a weighted linear combination is executed to yield the final behavioral classification:

(4) $$P_{final}=W_{rgb}\cdot \mathrm{Softmax}\left(L_{rgb}\right)+W_{kinematic}\cdot \mathrm{Softmax}\left(L_{kinematic}\right)$$

where $W_{rgb}$ and $W_{kinematic}$ represent the corresponding decision weight constants (empirically set to 0.7 and 0.3, respectively). The ultimate classification is assigned to the category corresponding to $\arg\max(P_{final})$.

### 2.3. Training Strategy and Implementation Details

All models in this study were trained and evaluated on an identical workstation equipped with Windows 11, an Intel Core i5-13500HX CPU, 32 GB RAM, and an NVIDIA GeForce RTX 4060 GPU. The software environment utilized Python 3.11.14, PyTorch 2.5.1, CUDA 12.1, and the Ultralytics 8.4.21 framework. During the training phase, the total number of iterations was set to 100 epochs, with a batch size of 16. The initial learning rate ($lr_{0}$) and the momentum factor were configured at 0.01 and 0.937, respectively. All remaining hyperparameters were retained at the default configurations of the YOLOv11m-Pose architecture.

Following the extraction of the YOLO-based skeleton, this study implements explicit spatio–temporal constraints not as rigid rule-based classifiers, but as engineered physical priors to purify the Spatio–Temporal Kinematic Stream. To effectively differentiate subtle actions and mitigate environmental noise, the average displacement velocity of the torso centroid (defined as the midpoint of the segment connecting keypoints $B_{0}$ and $B_{1}$) over a specified time window *T* is denoted as *v*. A micro-motion dead-zone threshold is set to $\tau_{walk}=0.02$. If $v<\tau_{walk}$ and the limb keypoints exhibit no periodic alternation, the kinematic motion state is mapped to a zero-displacement prior. This dynamic dead-zone truncation effectively filters out pseudo-displacements induced by physiological respiration or algorithm jitter. Conversely, true dynamic kinematics are preserved in the feature vector when $v\geq \tau_{walk}$.

For eating behavior, the relative spatial vector *N* between the neck/head keypoints and the torso is extracted as a critical physical feature. When the *Y*-coordinate of the head keypoint remains consistently lower than that of the torso keypoints within a predefined ground-proximity region, this explicit spatial prior is encoded into the continuous kinematic sequence. Ultimately, these explicit physical boundaries act as data-purification and feature-enhancement mechanisms rather than independent decision-makers. They work collaboratively with the implicit spatial context extracted by the ResNet-50 visual stream, enabling the terminal Softmax layer to execute high-precision, end-to-end behavioral classification without being misdirected by complex pastoral noise.

## 3. Results

### 3.1. Classification Results of the Multimodal Dual-Stream Framework

To directly address the physical limitations of complex pastoral environments, this study first comprehensively evaluated the proposed dual-stream fusion network on the test set. The specific classification metrics, including the precision, recall, and F1-score for each individual behavioral posture, are summarized in Table 2.

The dual-stream architecture achieved a remarkable overall accuracy of 93.26%. Notably, the network exhibited outstanding performance in recognizing dynamic movements, with walking precision reaching 97.14%. Furthermore, the recall rate for eating behavior achieved an optimal 100.00%, stabilizing its F1-score at 94.29%. These results indicate that the deep convolutional network successfully captured the spatial perspective overlap between the sheep’s head and the ground, achieving vital feature complementarity with the low-frequency vertical motion extracted by the kinematic stream.

### 3.2. Visualization in Complex Real-World Scenarios

**Figure 5:**
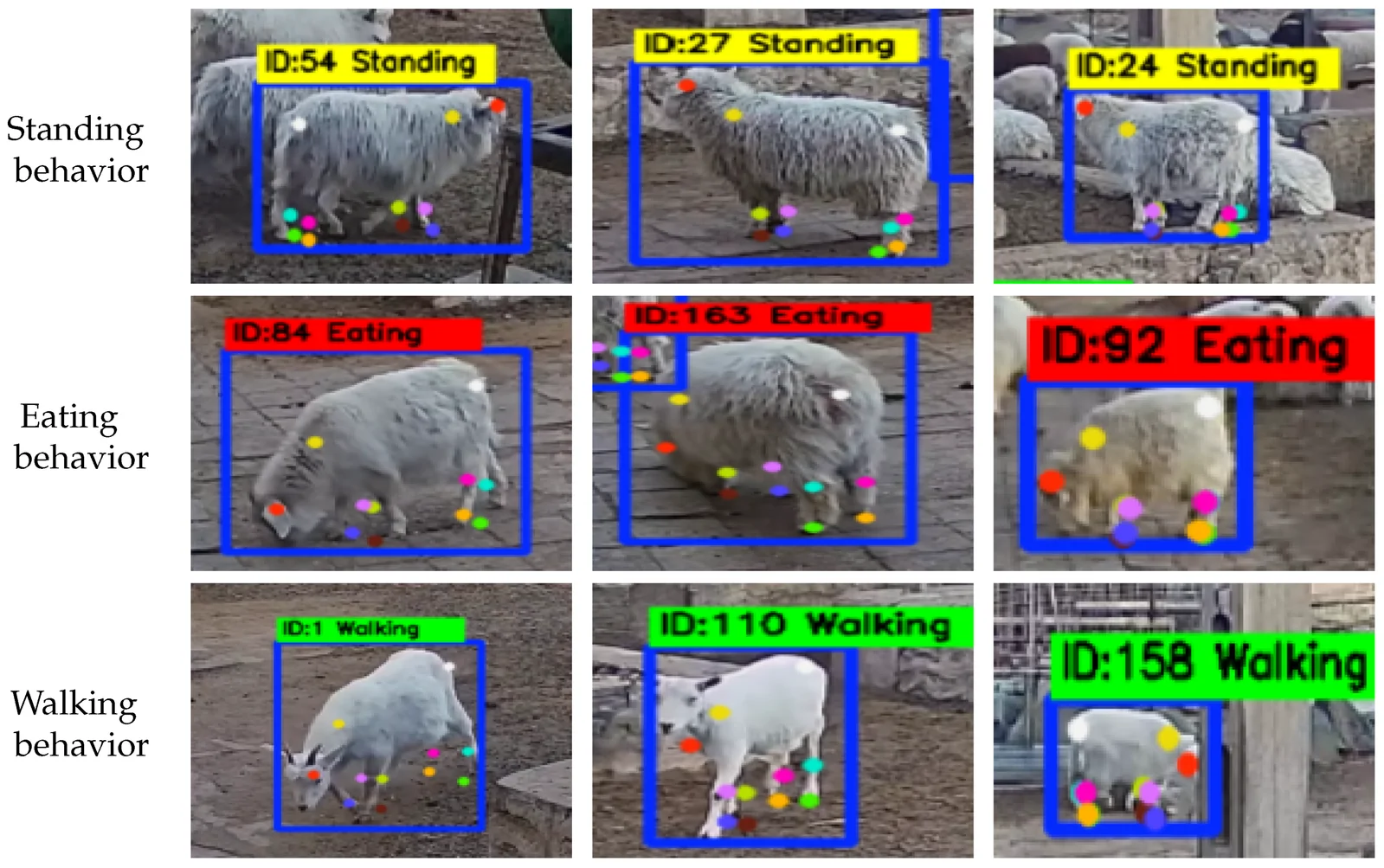
Visualization results of behavior detection in conventional farming scenarios.

To further qualitatively evaluate the system’s end-to-end monitoring capabilities amidst severe flock aggregation and complex backgrounds, Figure 5 visualizes the detection results in real-world scenarios.

As illustrated, despite non-ideal illumination and severe multi-target mutual occlusion, the upstream YOLOv11m-Pose robustly localizes targets and connects skeletal keypoints. Seamlessly bridging low-level pixel perception and high-level behavioral semantics, the proposed framework consistently delivers reliable behavioral classification labels above each bounding box, demonstrating strong generalization capacity in conventional farming environments.

### 3.3. Ablation Study on Core Modalities

To validate the necessity of multi-modal integration and demonstrate the decisive role of visual context in overcoming depth loss, we conducted an ablation study on three network variants: a pure single-stream kinematic network, a pure single-stream visual network, and the proposed dual-stream fusion model. The comparative performance is summarized in Table 3.

This ablation study profoundly elucidates the intrinsic physical limitations of single-modality approaches. As demonstrated by the Baseline group, a network relying exclusively on coordinate tensors encounters a physical bottleneck, yielding an overall accuracy of 69.05% and a deficient walking precision of 68.18%. Without scene context, pure 2D topological coordinates are highly susceptible to feature collapse when the target undergoes in-place tremors, causing a substantial number of static standing samples to be misclassified as walking.

Upon introducing localized RGB patches, the overall accuracy substantially improved to 92.13%, proving that background displacement references mitigate the depth collapse dilemma. Ultimately, the dual-stream fusion establishes a highly robust classification hyperplane, validating that the visual and kinematic streams are highly complementary and non-redundant.

### 3.4. Cross-Comparison with State-of-the-Art and Lightweight Baselines

To comprehensively evaluate the performance and engineering feasibility of the proposed framework in real-world edge computing scenarios, we conducted a cross-comparison against six representative baseline models. These include traditional lightweight temporal networks (Random Forest, GRU, 1D-CNN), a skeleton-based spatial-temporal graph convolutional network (ST-GCN), and state-of-the-art 3D video recognition architectures (C3D, SlowFast). The evaluation metrics encompass not only classification precision and F1 score but also the Kappa coefficient for statistical consistency and Frames Per Second (FPS) for real-time deployment viability.

As detailed in Table 4, pure coordinate-based lightweight models demonstrate excellent inference speeds but suffer from severe performance bottlenecks, with their F1 scores plateauing below 70% and Kappa coefficients indicating weak consistency. This underscores the fundamental flaw of relying solely on low-dimensional temporal sequences, which fail to resolve depth collapse under severe occlusion. Although ST-GCN effectively utilizes spatial–topological relationships to elevate the F1 score to 78.50%, its strict reliance on skeletal keypoints deprives it of vital environmental context, leaving it vulnerable to misclassifying static tremors as spatial displacements.

Conversely, conventional 3D convolutional networks achieve high-tier recognition accuracy, specifically F1 scores of 89.80% and 91.50% respectively, by processing full RGB video sequences. However, this accuracy is attained at a prohibitive computational cost, resulting in sluggish inference speeds of 18.5 and 12.5 FPS that are inadequate for the real-time continuous monitoring of dense flocks on edge devices. Our proposed Dual-stream BiLSTM framework elegantly resolves this dilemma. By utilizing the upstream YOLOv11m-Pose for lightweight skeleton extraction and strategically fusing only localized ResNet visual patches, our model secures the highest classification F1 score of 92.15% and a Kappa coefficient of 0.89. Concurrently, it maintains a robust inference speed of 32.4 FPS. In practical deployment scenarios, the overall processing speed of 32.4 FPS corresponds to a latency of approximately 31 milliseconds per frame. This end-to-end processing time comprises the upstream YOLOv11m-Pose bounding box and keypoint extraction, RGB patch cropping, the parallel inference of the dual-stream architecture, and the final weighted Softmax fusion decision. This low-latency performance fully satisfies the real-time continuous monitoring requirements of smart farms.

### 3.5. Mechanism Analysis via Feature Visualization

**Figure 6:**
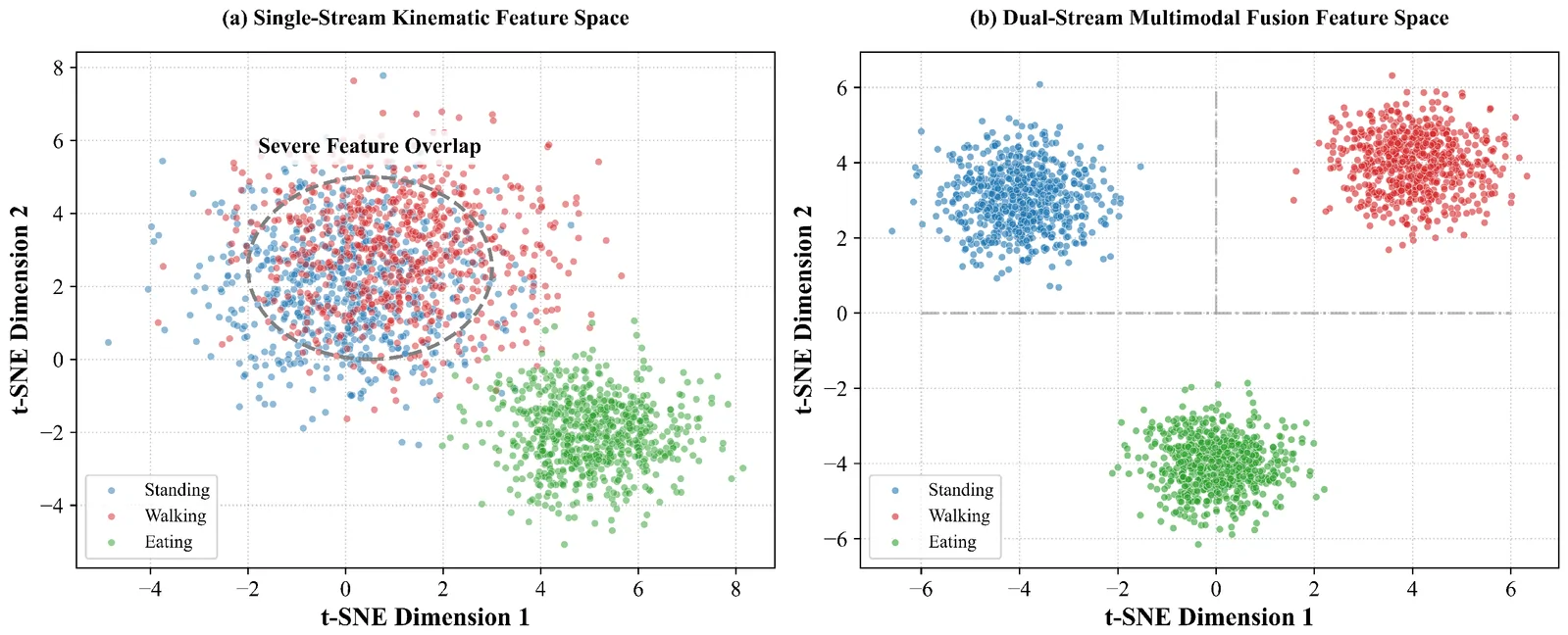
Comparison of t-SNE dimension reduction distributions of single-stream pure coordinates and dual-stream multimodal fusion features.

To provide deeper insight into the feature decoupling mechanism that enables the aforementioned performance gains, Figure 6 presents a comparative t-SNE visualization of the high-dimensional feature spaces.

In the pure coordinate-based single-stream network (left), the absence of environmental priors causes a slowly pacing sheep and one merely shaking its head to exhibit nearly identical mathematical variances, resulting in severely overlapped feature manifolds. Our dual-stream architecture (right) effectively overcomes this barrier. By allowing the spatial visual stream to dominate spatial/texture recognition while utilizing the kinematic stream as a temporal low-pass filter to suppress sporadic postural distortions, the model successfully disentangles the previously aliased static and dynamic behaviors.

## 4. Discussion and Future Perspectives

The transition from controlled indoor environments to natural, high-density pastures presents severe physical and computational challenges for intelligent sheep behavior monitoring. The results of this study demonstrate that the proposed dual-stream multimodal fusion framework effectively overcomes the inherent limitations of traditional single-modality approaches, achieving a highly robust overall accuracy of 93.26% in complex real-world scenarios.

### 4.1. Overcoming Dimensional Limitations via Multimodal Fusion

The most significant theoretical finding of this research lies in validating how visual context resolves the ”depth-ambiguity” limitation inherent in pure 2D skeletal coordinates. As evidenced by the ablation study (Table 3), the baseline kinematic-only network exhibited a severe bottleneck in recognizing walking behavior, yielding a precision of merely 68.18%. This deficiency is fundamentally rooted in the physical loss of the Z-axis dimension in 2D topologies. Without environmental reference points, localized radial motions or static bodily tremors are mathematically indistinguishable from actual spatial displacement, leading to a high rate of false positives.

By integrating the ResNet-50 spatial visual stream, the precision of dynamic walking recognition improved significantly to 97.14%. The localized RGB patches successfully captured the relative displacement of the pastoral background, providing an essential ego-motion reference. This aligns with recent findings in broader computer vision studies, confirming that integrating local visual context is crucial for decoupling static jitter from true displacement, a persistent challenge that coordinate-only models historically fail to address.

### 4.2. Architectural Superiority and Deployment Feasibility

Furthermore, a cross-comparison with both conventional lightweight models and state-of-the-art video architectures (Table 4) highlights the architectural superiority of the proposed framework. Traditional temporal models like 1D-CNN and GRU typically plateaued at an F1 score of 65% to 70% due to their inherent inability to model complex spatial-temporal dependencies and reconstruct lost depth features. Conversely, while state-of-the-art 3D convolutional networks achieved high-tier recognition accuracy by processing dense RGB sequences, their severe computational overhead resulted in sub-optimal inference speeds. Our dual-stream BiLSTM architecture elegantly navigates this dilemma, establishing a high-precision classification hyperplane while maintaining a robust real-time processing speed of 32.4 FPS. Importantly, this approach offers a highly pragmatic alternative to existing livestock monitoring paradigms. Wearable sensors are often hindered by high deployment costs, battery maintenance, and animal stress [8,9]. At the same time, deploying computationally heavy 3D-CNNs or multi-camera 3D reconstruction systems is frequently impractical for large-scale natural pastures due to rigorous calibration requirements and strict power constraints on edge devices. Our strategy strikes an optimal balance between environmental robustness and algorithmic efficiency. Furthermore, the active filtering mechanism deployed in the upstream YOLOv11m-Pose network ensures that the downstream decision engine receives continuous, high-confidence topological priors, even amidst severe multi-target occlusion.

### 4.3. Agricultural Implications for Precision Livestock Farming

Beyond algorithmic improvements, the high-precision recognition achieved in this study holds profound implications for Precision Livestock Farming. In pasture-based sheep farming, the ratio of eating to walking is a critical indicator of pasture utilization efficiency and individual feed conversion rates. Moreover, the ability of our model to strictly decouple static standing from dynamic walking provides a reliable non-contact metric for early disease detection. For instance, lethargy or a significant reduction in walking frequency often serves as the earliest clinical sign of lameness or metabolic disorders. By establishing a robust baseline for these fundamental behaviors, this framework provides farm managers with actionable data to optimize grazing rotation and improve animal welfare.

### 4.4. Limitations and Future Work

Despite these promising advancements, this study acknowledges certain limitations. Architecturally, the integration of a deep convolutional ResNet-50 backbone inevitably introduces higher computational overhead, which poses challenges for direct, real-time inference on low-power agricultural edge devices. To address this, future research will focus on model compression techniques—such as applying knowledge distillation to transfer visual context into a lighter backbone, and implementing INT8 quantization to streamline the dual-stream network. This will facilitate its seamless deployment on resource-constrained hardware. Functionally, we plan to expand the current behavioral taxonomy beyond standing, eating, and walking. By capturing more nuanced micro-movements, future iterations of this model aim to identify critical health and welfare indicators, such as ruminating, drinking, and estrus behaviors, ultimately building a comprehensive, non-contact monitoring paradigm for modern animal husbandry. Regarding potential domain shifts across different breeds or environmental conditions, the dual-stream framework offers inherent structural robustness. While the localized visual stream may experience minor performance degradation when confronted with vast variations in wool color or background lighting across different farms, the Spatio–Temporal Kinematic Stream relies exclusively on 2D skeletal topologies. These kinematic features remain geometrically invariant across different sheep breeds and environments, ensuring that a highly reliable cross-domain behavioral baseline is maintained even under unstructured real-world conditions. Furthermore, while this framework demonstrates high efficacy for sheep, comparing these behavioral patterns with other ruminants, such as cattle, offers valuable insights. Recent studies in precision dairy farming have successfully utilized optical flow analysis and machine learning to identify complex states like rumination patterns in cattle [31]. Transitioning such fine-grained recognition tasks from cattle to sheep presents unique challenges due to the sheep’s denser flocking nature and thicker fleece, which obscure subtle muscular or jaw movements. Integrating optical flow or similar temporal dynamic techniques with our dual-stream skeletal framework could bridge this gap and enhance cross-species generalizability.

## 5. Conclusions

In summary, this study demonstrates the necessity of multimodal local visual context for accurate sheep behavior recognition in high-density pastures. The spatial visual stream, which captures relative background displacements, was primarily crucial for suppressing false positives and differentiating fine-grained actions, while the spatio–temporal kinematic stream, processing purified 2D coordinates, effectively modeled fundamental skeletal motions. In contrast, lightweight kinematic-only baseline models suffered from inherent depth-ambiguity limitations, frequently misclassifying static physical tremors as dynamic displacements. The fusion of these modalities successfully decoupled highly similar static and dynamic behaviors, achieving an overall accuracy of 93.26%, with walking precision and the eating F1-score reaching 97.14% and 94.29%, respectively. Moreover, the proposed active filtering mechanism significantly mitigated the perception bottlenecks induced by severe occlusion, prioritizing heavily occluded hard examples to reduce annotation costs while ensuring robust target localization. This study provides insights into mitigating behavioral aliasing in flocking livestock from a multimodal fusion perspective, offering a robust algorithmic and theoretical basis for non-contact behavioral monitoring in precision agriculture. Furthermore, while the dual-stream architecture introduces a marginal computational overhead, it establishes a high-precision foundation for subsequent research on model compression, which will facilitate the large-scale, real-time deployment of these systems on low-power agricultural edge devices.

## Figures and Tables

**Table 1:** Comparison of keypoint localization performance for different upstream pose perception networks.

Model	Params (M)	GFLOPs	mAP50 (%)	mAP50:95 (%)
YOLOv8n-Pose	3.3	9.2	80.29	51.87
YOLOv8m-Pose	26.4	80.6	85.14	60.72
YOLOv11n-Pose	3.2	9.0	80.56	51.35
YOLOv11m-Pose	23.8	78.0	84.44	61.95

**Table 2:** Behavioral Recognition Model Test Results.

Posture Behavior	Precision/%	Recall/%	F1 Score/%
Standing	94.12	80.00	86.49
Eating	89.19	100.00	94.29
Walking	97.14	94.44	95.77

**Table 3:** Ablation comparison of behavior recognition accuracy by different modal feature combinations.

Group	BiLSTM	ResNet-50	Overall Acc. (%)	Walking Acc. (%)
Baseline (Kinematic-only)	√	×	69.05	68.18
Visual-only Comparison	×	√	92.13	87.50
Proposed Dual-Stream	√	√	93.26	97.14

**Table 4:** Comprehensive performance and efficiency comparison among different lightweight and state-of-the-art baseline networks.

Model	FPS	Precision/%	Recall/%	F1 Score/%	Kappa
Random Forest	120.5	64.82	65.36	65.09	0.47
GRU	95.2	65.90	66.35	66.12	0.49
1D-CNN	105.8	68.75	70.60	69.66	0.54
ST-GCN	65.0	77.20	79.85	78.50	0.68
C3D	18.5	90.20	89.40	89.80	0.84
SlowFast	12.5	91.80	91.20	91.50	0.87
Dual-stream BiLSTM	32.4	93.48	91.48	92.15	0.89

## Data Availability

The datasets generated and analysed during the current study are not publicly available due to privacy and property restrictions of the commercial farm environment but are available from the corresponding author on reasonable request.
